# Elevated Serum Levels of Inflammation-Related Cytokines in Mild Traumatic Brain Injury Are Associated With Cognitive Performance

**DOI:** 10.3389/fneur.2019.01120

**Published:** 2019-10-23

**Authors:** Yingxiang Sun, Lijun Bai, Xuan Niu, Zhuonan Wang, Bo Yin, Guanghui Bai, Danbin Zhang, Shuoqiu Gan, Chuanzhu Sun, Shan Wang, Feng Zhu, Ming Zhang

**Affiliations:** ^1^Department of Medical Imaging, The First Affiliated Hospital of Xi'an Jiaotong University, Xi'an, China; ^2^The Key Laboratory of Biomedical Information Engineering, Ministry of Education, Department of Biomedical Engineering, School of Life Science and Technology, Xi'an Jiaotong University, Xi'an, China; ^3^Department of Neurosurgery, The Second Affiliated Hospital and Yuying Children's Hospital of Wenzhou Medical University, Wenzhou, China; ^4^Department of Radiology, The Second Affiliated Hospital and Yuying Children's Hospital of Wenzhou Medical University, Wenzhou, China; ^5^Center for Translational Medicine, The First Affiliated Hospital of Xi'an Jiaotong University, Xi'an, China

**Keywords:** inflammation-related cytokines, post-concussion symptoms, cognitive performance, mild traumatic brain injury, follow-up

## Abstract

Mild traumatic brain injury (mTBI) is the most common neurological insult and leads to long-lasting cognitive impairments. The immune system modulates brain functions and plays a key role in cognitive deficits, however, the relationship between TBI-induced changes in inflammation-related cytokine levels and cognitive consequences is unclear. This was investigated in the present study in two cohorts of individuals within 1 week of mTBI (*n* = 52, *n* = 43) and 54 matched healthy control subjects. Patients with mTBI were also followed up at 1 and 3 months post-injury. Measures included cognitive assessments and a 9-plex panel of serum cytokines including interleukin (IL)-1β, IL-4, IL-6, IL-8, IL-10, IL-12, chemokine ligand 2 (CCL2), interferon-γ (IFN-γ), and tumor necrosis factor-α (TNF-α). The contribution of cytokine levels to cognitive function was evaluated by multivariate linear regression analysis. The results showed that serum levels of IL-1β, IL-6, and CCL2 were acutely elevated in mTBI patients relative to controls; CCL2 level was remained high over 3 months whereas IL-1β and IL-6 levels were declined by 3 months post-injury. A high level of CCL2 was associated with greater severity of post-concussion symptoms (which survived in the multiple testing correction); elevated IL-1β was associated with worse working memory in acute phase (which failed in correction); and acute high CCL2 level predicted higher information processing speed at 3 months post-injury (which failed in correction). Thus, acute serum cytokine levels are useful for evaluating post-concussion symptoms and predicting cognitive outcome in participants with mTBI.

## Introduction

Traumatic brain injury (TBI) is a public health burden and its incidence ranging from 106 to 790 per 1,00,000 people worldwide yearly (1). About 70–90% of TBI are classified as mild TBI (mTBI) (2), which is associated with long-term cognitive impairment including deficits in attention, working memory and executive function. Owing to the heterogeneous pathology of mTBI, there are few effective treatments (3, 4). Guided by preclinical studies partly, therapeutic strategies for mTBI have targeted in protein aggregation, inflammation, metabolic disruption, cell proliferation, and neurotransmitter signaling, but these have had limited success. Clarifying the pathogenic changes underlying cognitive impairment following mTBI can lead to the development of more effective treatment.

Inflammation played a crucial role in cognitive deficits (5–7); for instance, an elevated level of interleukin (IL)−6 was found to be associated with impaired executive function following stroke (8). And has also been implicated in Alzheimer's disease, serum IL-6 is negatively correlated with general cognitive functioning (9). A robust inflammatory response is induced in the injured brain and peripheral circulatory system following a traumatic impact (10), that can persist for many months (11). Inflammation-related cytokines propagate the inflammatory response, promote excitotoxicity, and oxidative injury giving rise to the neurotoxicity (10, 12). On the other hand, neuroinflammation attenuates central nervous system (CNS) damage through angiogenic, neurotrophic, and other mechanisms (13, 14). However, mTBI-induced changes of inflammatory cytokines and its association with cognitive consequences have not been investigated.

Cytokine profiles have been linked to adverse events and poor global outcomes after moderate to severe TBI (15, 16). For example, in children with TBI, IL-1β level in the first 24 h was negatively correlated 6 months later with Glasgow Outcome Scale (GOS) score (17). Previous studies investigating the functional significance of cytokine levels after mTBI have largely been cross-sectional in design (18), and have provided limited information. Indeed, little is known about the effects of mTBI-induced changes in inflammatory cytokine levels on the recovery of cognitive function. As such, there is a need for a systematic, prospective longitudinal study that can address this point.

To this end, the present study investigated the changes in serum levels of cytokines shortly after mTBI (<7 days) and 3 months later and the association between cytokine profiles and cognitive deficits. We further calculated a cytokine load score (CLS) as a measure of overall inflammatory burden to assess the predictive value of inflammation for cognitive outcome following mTBI (19, 20).

## Materials and Methods

### Participants

Consecutive patients who underwent non-contrast head computed tomography after acute head trauma at local emergency department between August 2016 and June 2017 constituted Cohort 1; these patients were followed-up at 1 and 3 months post-injury so that their recovery could be monitored. Cohort 2 comprised patients recruited from August 2017 to December 2017; there were no follow-up of this group.

Inclusion criteria for mTBI patients were based on those outlined by the World Health Organization Collaborating Centre for Neurotrauma Task Force (21): (i) initial Glasgow Coma Scale score of 13–15; (ii) one or more of any following: loss of consciousness <30 min, post-traumatic amnesia <24 h and/or other transient neurological abnormalities such as focal symptoms and seizure; and (iii) diagnosed within 1 week of having experienced mTBI. Exclusion criteria were as follows: (i) pre-TBI, neurological or psychiatric illness diagnosed prior to TBI; (ii) drug or alcohol addiction; and (iii) mTBI occurring as a complication of other injuries (e.g., systemic, facial injuries, or spinal cord injury) or other problems (e.g., psychological trauma, language barrier, or coexisting medical conditions), or caused by penetrating craniocerebral injury.

Cohort 1 comprised 52 patients; blood samples were collected within 7 days post-injury (T1: 2.63 ± 1.23 days, all presented as Mean ± SD) and at 1-month (T2: 38.56 ± 9.33 days post-injury) and 3-months (T3:110.58 ± 21.13 days post-injury) follow-ups, reflecting the time course of recovery following mTBI (22). Neuropsychological tests were performed within 24 h of blood sampling. Cohort 2 comprised 43 mTBI patients within 7 days post-injury (T1: 3.16 ± 1.74 days).

In addition, 54 age-, gender-, and education level-matched healthy control subjects were recruited according to same set of exclusion criteria as those applied to mTBI patients. All participants provided written, informed consent for their participation and the study was approved by a local institutional review board and carried out in accordance with the tenets of the Declaration of Helsinki.

### Serum Biomarker Detection

Serum samples were collected in the morning at 07:00–08:00 h and centrifuged, and aliquots of supernatant were stored at −80°C until analysis. Serum cytokine levels (pg/ml) were measured using reagents on a Luminex multiplex bead system (Luminex Austin, TX, USA). A fluorescence detection laser optic system was used to simultaneously detect binding of each individual protein onto microspheres, thereby allowing analysis of several analytes in a single sample. Intra- and inter-assay coefficients of variation for Luminex quantification were <20 and 25%, respectively. Samples with levels that were undetectable by the assay were set to 0.01 pg/ml. The criteria for the choice of cytokines were mainly based on whether it's associated with TBI or clinical symptoms such as PCS and cognitive function in previous studies (7, 16, 23). According to their effects on inflammation, cytokines we selected can be grouped as (i) the archetypal pro-inflammatory cytokines: IL-1β, IL-6, and IL-12, and the anti-inflammatory cytokines IL-4, IL-10; (ii) chemokine (C-C motif) ligand 2 or monocyte chemoattractant protein-1(CCL2 or MCP-1) and member of the CXC chemokine family (CXCL8) IL-8; (iii) interferon-γ (IFN-γ); and (iv) tumor necrosis factor α (TNF-α).

### Neuropsychological Tests

Cognitive testing were performed within 24 h of blood sample collection and included (i) Trail-Making Test Part A (TMA) and Digit Symbol Coding score (DSC) from the Wechsler Adult Intelligence Scale III (WAIS-III) to measure cognitive information processing speed (24); (ii) Forward Digit Span and Backward Digit Span from the WAIS-III to evaluate working memory (25); (iii) Verbal Fluency Test to assess verbal fluency including language ability, semantic memory and executive function (26). And post-concussive symptoms (PCS) were evaluated with the Rivermead Post-Concussion Symptom Questionnaire (RPQ) (27). The tests were administered in face-to-face interviews by two psychologists blinded to the nature of the study.

### Statistical Analysis

Statistical analyses were performed using the SPSS v.21 (IBM Corp, Armonk, NY, USA) and Prism v.5 (GraphPad Inc., La Jolla, CA, USA). The independent two-sample *t*-test and Mann Whitney test were used to evaluate differences between mTBI and control groups in acute phase based on the assumption of data normality. The chi-square test was used to evaluate categorical variables. General linear model was used to compare cognitive performance between patients and controls after adjusting for age and education level. Statistical significance was defined by an unpaired, two-tailed *P* < 0.05, except for an adjusted *P* < 0.0056 (0.05/9) for comparisons of the nine cytokines with Bonferroni correction.

Due to the skewed distribution of data, Friedman test was used to examine changes in cytokines levels, CLS and neuropsychological scores as a function of recovery after mTBI at three time points. The Bonferroni *post-hoc* correction for multiple comparisons was applied, yielding an adjusted *P* < 0.0167 (0.05/3) for three time points in mTBI group; this was compared to the values in the control group with Mann-Whitney test with Bonferroni correction, yielding an adjusted *P* < 0.0167 (0.05/3). In addition, percentage changes of cytokine levels, CLS and neuropsychological scores from acute phase over during of follow ups were determined by (T2-T1)/T1 and (T3-T1)/T1.

Inflammation-related cytokines showing significant inter-group differences in acute phase were used to calculate the CLS (28). Cytokine levels in the control group were divided into deciles; patients and control subjects with values higher than the 90th decile or lower than 10th decile were assigned values of “10” and “1,” respectively. The values for all cytokines were then summed to obtain CLS. The capacity of cytokines and CLS to distinguish patients from controls was assessed by generating a receiver operating characteristic (ROC) curve and calculating area under the curve (AUC).

Stepwise multivariate linear regression analysis was carried out to determine the association between elevated cytokine levels and CLS in acute phase and neuropsychological scores at different time points following mTBI. The cytokines levels or CLS, along with age, gender and number of years of education were introduced stepwise into model as independent variables, with neuropsychological test scores as dependent variables. The criterion for entry or removal of a variable was *F*-value of 0.05. The multiple linear regression analysis was based on the following assumptions: the relationship between independent and dependent variables was linear; errors between independent and dependent values were normally distributed; and no multicollinearity was found. Given the multiple cognitive tests at different time points and the use of various non-independent measures in the tests, we calculated separate alpha thresholds in each of the main analyses to decrease the probability of type II errors (29). The significance threshold of associations between PCS and cytokine levels or CLS was set at *P* < 0.0167 (three time points = three tests). The significance threshold of associations between cognitive test score and cytokine levels or CLS was set at *P* < 0.003 (three time points × five cognitive tests = 15 tests).

## Results

### Characteristics of the Study Population

There were no significant differences between mTBI Cohorts 1 and 2 in terms of clinical characteristics (Supplementary Table S1). Data from Cohort 2 (*n* = 43) and Cohort 1 (*n* = 52) were therefore combined, yielding a pooled sample of 95 patients in acute phase. The clinical characteristics of 95 patients are presented in Table 1. There were no differences in age, gender, and education level between mTBI patients and controls subjects. The most common cause of injuries was acceleration/deceleration caused by accident (60/95, 63.2%), followed by assaults (21/95, 22.1%), ground-level fall (7/95, 7.4%), fall from height (6/95, 6.3%), and direct impact blow to head (1/95, 1.1%).

**Table 1:** Summary of demographic and clinical information for mTBI patients and normal control subjects.

**Demographic^a^**	**mTBI (*n* = 95)**	**Controls (*n* = 54)**	***P*-value**
Age in years	35.93 ± 13.69	35.74 ± 11.51	0.987
	(33.14–38.71)	(32.60–38.88)	
Gender	55M:40F	29M:25F	0.731
Education in years	8.51 ± 3.75	9.43 ± 4.14	0.103
	(7.74–9.27)	(8.30–10.55)	
**Neuropsychological tests^a^**			
Trail Making A	62.32 ± 45.93	41.70 ± 23.55	**0.012**
	(52.96–71.67)	(35.27–48.13)	
Digit symbol coding	34.91 ± 15.85	46.93 ± 16.88	**<0.001**
	(31.68–38.13)	(42.32–51.53)	
Digit span-forward	7.84 ± 1.53	8.33 ± 1.67	0.653
	(7.53–8.15)	(7.87–8.79)	
Digit span-backward	3.79 ± 1.34	4.51 ± 1.90	0.233
	(3.51–4.06)	(4.00–5.04)	
Language fluency	16.14 ± 5.16	18.93 ± 6.51	0.109
	(15.09–17.19)	(17.15–20.70)	
**Symptoms severity^a^**			
PCS	10.27 ± 7.26	2.33 ± 2.83	**<0.001**
	(8.79–11.75)	(1.56–3.11)	
**mTBI severity** ***n*** **(%)**			
Loss of conscious	86 (90.5%)	NA	
Post-traumatic amnesia	9 (9.5%)	NA	
GCS = 15	95 (100%)	NA	
GCS = 13, 14	0 (0%)	NA	
**Causes for mTBI** ***n*** **(%)**			
Acceleration/deceleration	60 (63.2%)	NA	
Ground level fall	7 (7.4%)	NA	
Fall from height	6 (6.3%)	NA	
Assaults	21 (22.1%)	NA	
Direct impact blow to head	1 (1.1%)	NA	

a *Continuous variables are expressed as mean ± SD (90% confidence intervals) and categorical variables are expressed as a frequency and percentage. Results of neuropsychological tests are presented as raw scores. Statistically significant P values are shown in bold. GCS, Glasgow Coma Scale; mTBI, mild traumatic brain injury; NA, non-available; PCS, Post-concussive symptoms*.

### Neuropsychological Measures

Patients reported significant discomfort in post-concussive symptoms (PCS) on scale of the RPQ (*P* < 0.001). There was worse performance in information processing speed assessed by the Digit Symbol Coding task (DSC) (*P* < 0.001), Trail-Making Test Part A (TMA) (*P* = 0.012) relative to controls after adjusting for age and education level. Besides, patients in Cohort 2 showed worse performance in Language Fluency test compared with control subjects (*P* = 0.018) (Table 1).

A longitudinal analysis of Cohort 1 showed that the main effects of time were significant for PCS (χ^2^ = 14.241, *P* = 0.001), TMA scores (χ^2^ = 19.240, *P* < 0.001) and DSC scores (χ^2^ = 38.821, *P* < 0.001), suggesting that PCS and cognitive function improved with time after mTBI. A *post-hoc* analysis revealed significant recovery in the TMA and DSC at T2 (TMA, *P* = 0.013; DSC, *P* = 0.006) and T3 (TMA, *P* < 0.001; DSC, *P* < 0.001) compared with T1, with the scores reaching control level at T2 (both *P* > 0.0167). Median of percentage changes (interquartile range) of TMA and DCS from T2 to T1 were −17.35% (43%) and 12.55% (42%), and from T3 to T1 were −22.85% (42%) and 19.68% (59%), respectively. However, although PCS was improved at T2 (*P* = 0.002) and T3 (*P* = 0.001) compared with T1, they were more severe than in controls at T3 (*P* < 0.001); median of percentage changes of PCS in T2 and T3 were −48.08% (71%) and −47.22% (100%) (Supplementary Figures S1, S2).

### Serum Cytokine Levels and Determination of CLS

**Figure 1:**
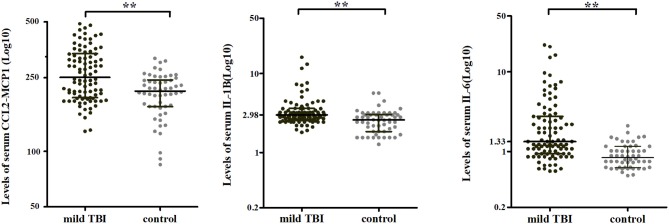
Serum cytokine levels in mTBI patients and controls in acute phase. Serum levels of CCL2, IL-1β, and IL-6 were higher in mTBI patients (black, *n* = 95) than in controls (gray, *n* = 54). Horizontal bars represent the medians and interquartile range in pg/ml. **P < 0.001.

Serum levels of CCL2, IL-1β, and IL-6 in acute phase were higher in mTBI patients than in controls (all *P* < 0.001) after Bonferroni correction with an adjusted *P* < 0.0056 (0.05/9 for nine cytokines; Figure 1 and Supplementary Table S2; given that the values were not normally distributed, thus presented as median and interquartile ranges). AUCs calculated from ROC curves of each cytokine ranged from 0.070 to 0.078, which distinguished mTBI patients from control subjects (Supplementary Figure S3).

**Figure 2:**
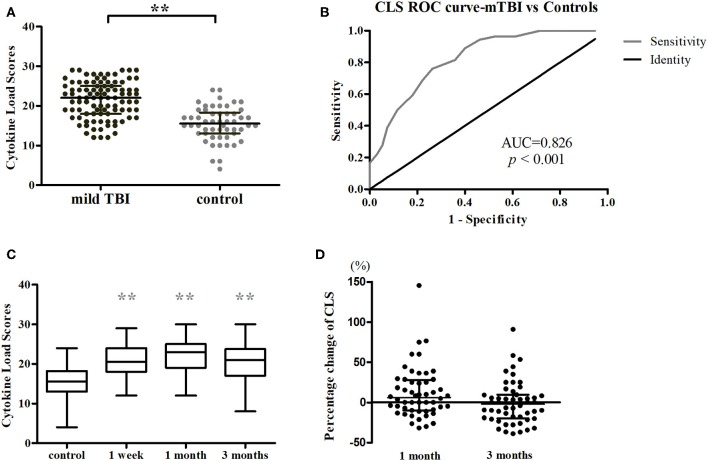
Analysis of CLSs. **(A)** CLS was higher for mTBI patients (*n* = 95, black) than control subjects (*n* = 54, gray) in acute phase. **P < 0.001. **(B)** CLS ROC curve. **(C)** Longitudinal changes in CLS after injury in Cohort 1 (*n* = 52 for all three time points). Horizontal bars represent the medians and interquartile range in pg/ml. Statistically significant differences between patients and controls at each time points and between different time points within patients are indicated by gray asterisks and black asterisks, respectively. **P < 0.001. **(D)** Percentage changes of CLS from acute phase to follow-ups. Boxplots represent medians and interquartile (*n* = 52 for both time points).

The cytokines that were significantly elevated in acute phase of mTBI (IL-1β, IL-6, and CCL2) were used to calculate CLS, which was higher in mTBI patients than in controls (*P* < 0.001) (Figure 2A). AUC calculated from ROC curve of CLS was able to discriminate between the mTBI and control groups (AUC = 0.83, 95% confidence interval = 0.76–0.89, *P* < 0.001; Figure 2B).

**Figure 3:**
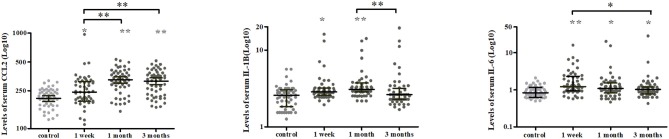
Temporal profiles of serum cytokine levels over a 3-months period in mTBI patients. Scatter plots showing a higher CCL2 at 1 and 3 months while lower IL-1β and IL-6 levels at 3 months post-injury in mTBI patients. Horizontal bars represent the median and interquartile range in pg/ml (*n* = 52 for all three time points). Statistically significant differences between patients and controls at each time points and between different time points within patients are indicated by gray asterisks and black asterisks, respectively. *P < 0.005 and **P < 0.001.

**Figure 4:**
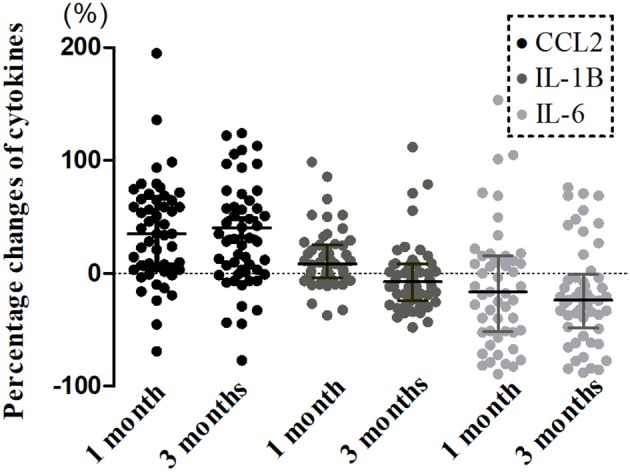
Percentage changes from acute phase to follow-ups in levels of serum cytokines in mTBI patients. Boxplots represent medians and interquartile (*n* = 52 for both time points).

A longitudinal analysis of Cohort 1 showed that the main effects of time were significant for CCL2 (χ^2^ = 25.239, *P* < 0.001), IL-1β (χ^2^ = 19.408, *P* < 0.001), IL-6 (χ^2^ = 9.169, *P* = 0.010), and CLS (χ^2^ = 7.320, *P* = 0.026; Figures 2C, 3). In *post-hoc* analysis, CCL2 level was higher at T2 (*P* < 0.001) and T3 (*P* < 0.001) relative to T1, and median of percentage changes (interquartile range) were 35.04% (62%) and 29.37% (69%) in T2 and T3 (Figure 4); IL-1β showed no significant change between T1 and T2 (*P* = 0.011) but was decreased at T3 compared with T2 (*P* < 0.001); and IL-6 level was lower at T3 compared with T1 (*P* = 0.003). Median of percentage changes for IL-1β and IL-6 were 8.17% (29%) and −13.78% (67%) in T2 and −8.07% (33%) and −23.07(50%) in T3 (Figure 4). CLS showed no significant differences among mTBI groups and its median of percentage changes were 6.07% (37%) and −2% (29%) in T1 and T2 (Figure 2D). Relative to control group, only IL-1β showed no difference at T3 (*P* = 0.34).

### Relationship Between Cytokine Levels and Neuropsychological Test Scores

**Figure 5:**
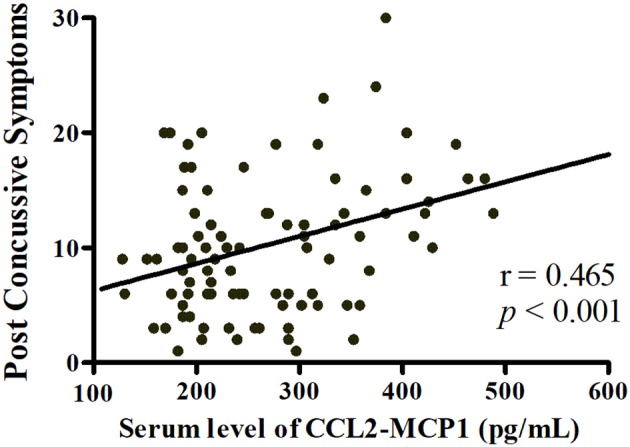
Linear regression analysis of the relationship between neuropsychological test performance and serum cytokine levels. Higher serum CCL2 level was associated with greater severity of post-concussive symptoms in acute phase post-injury (*n* = 95).

Stepwise multiple linear regression was performed; only one model including cytokines survived the multiple testing correction. In pooled analysis for acute phase (*n* = 95), a higher CCL2 level was associated with greater severity of PCS [CCL2, standardized β = 0.465, *P* < 0.001; overall model: *F*_(1, 93)_ = 24.22, *P* < 0.001, adjusted *R*^2^ = 0.198; Figure 5]. Elevated IL-1β tended to be associated with poorer working memory as determined by Digit Span backward test [IL-1β, standardized β = −0.187, *P* = 0.047; overall model: *F*_(2, 92)_ = 12.442, *P* < 0.001, adjusted *R*^2^ = 0.196], although this failed to pass correction.

In the follow up for Cohort 1 (*n* = 52), elevated CCL2 at T1 predicted a tendency for higher Digital Symbol coding test score at T3 [CCL2, standardized β = 0.214, *P* = 0.009; overall model: *F*_3, 48_ = 43.509, *P* < 0.001, adjusted *R*^2^ = 0.714]; however, the significance of coefficient of independent variables failed to pass the correction.

To determine whether cytokine levels influence the recovery of PCS and cognitive measures from acute to chronic phase of mTBI, we calculated the changes in these scores by subtracting T1 from T3 in Cohort 1 (*n* = 52). A change in working memory as evaluated by Digit Span backward test tended to be associated with IL-1β level in acute phase [IL-1β, standardized β = 0.336, *P* = 0.015; overall model: *F*_1, 50_ = 6.361, *P* = 0.015, adjusted *R*^2^ = 0.095], but this also failed to pass the correction.

## Discussion

In this study, serum cytokine levels were increased after mTBI and persisted from acute to chronic phase. Higher cytokine levels were associated with greater severity of PCS and worse working memory acutely. However, cytokine levels in acute phase showed a positive association with information processing speed at 3 months post-injury. This is the first study to investigate inflammatory cytokine profiles in mTBI patients and their relationship to cognitive dysfunction, which is a major factor contributing to poor prognosis in mTBI.

The inflammatory response to TBI is triggered by brain tissue damage and involves the activation of resident microglia, astrocytes, and peripheral inflammation (15, 16). Cytokines levels are elevated in acute phase after mTBI, but little is known about the changes in chronic phase (19, 30). In the present longitudinal study, we found that the levels of inflammation-related cytokines were elevated at 3 months post-injury, suggesting that low-grade systemic inflammation persists in mTBI patients, as suggested by a study of C-reactive protein (CRP) levels (31). Animal and clinical studies based on neuropathological and position emission tomography observation have reported that microglial activation can continue for more than 1 year after TBI (11, 32, 33). Thus, mTBI-associated neuroinflammation in the CNS and at peripheral sites is long-lasting.

PCS were evaluated with RPQ and included physical, emotional, behavioral, and cognitive symptoms (such as headache, anxiety, fatigue, irritability, memory, and concentration problems, etc.) (27). In our study, the level of the inflammatory cytokine CCL2 was positively associated with PCS, which is consistent with previous studies (23, 31, 34). For instance, elevated levels of CRP and S-100β (a glial cell protein) in acute phase were associated with increased risk of persistent PCS (31, 34). Systemic inflammation can trigger neuroinflammation through circumventricular organs, vagal afferents, or the brain endothelium (35), undermining the microstructural integrity of white matter (36, 37), disrupting microglia function in synaptic plasticity (38) and reducing cognitive functioning. Stimuli causing neuroinflammation can directly injure the trigeminal afferent nerves or the leptomeningeal or cerebrovascular structures that they innervated, leading to post-traumatic headaches (39). Peripheral inflammation activates the hypothalamic-pituitary-adrenal (HPA) axis (40), which resulting in chronic stress-associated anxiety and depression (41). In general, systemic inflammation contributes to PCS. A new term post-inflammatory brain syndromes (PIBS) has been proposed to encompass the contribution of systemic inflammation to the development of PCS-like symptoms, even in patients without head injury (23).

In the current study, IL-1β level tended to be negatively associated with working memory in acute phase and positively associated with changes in working memory at 3 months post-injury. The former can be ascribed to the role of IL-1β in mTBI-related cognitive impairment (42), whereas the latter could be due to a lower cognitive function at baseline, which would provide room for change compared with a normal level of inflammation that changes little over time.

Diffuse axonal injury caused by shear stress and tissue deformation often occurs in mTBI (43). Information processing speed depends on the integrity of the myelin sheath surrounding neuronal axonal fibers (44). During recovery from mTBI, secondary neuroinflammation contributes to white matter damage (45) and affects the speed of information processing. In our study, CCL2 level tended to be positively associated with information processing speed. Within hours of injury, astrocytes produce CCL2, the level of which is correlated with the number of recruited monocytes (46). The recruitment of peripheral monocytes to the meninges has been shown to exert beneficial effects on post-mTBI recovery by promoting meningeal remodeling and vascular repair (47).

In this study, we developed the CLS to reflect overall cytokine burden, which was more useful as a biomarker for mTBI than measurement of a single cytokine. It has been demonstrated that machine learning or principal component analysis of cytokine levels is more accurate for the diagnosis of mTBI and better predicts patient outcome than our approach (30, 48); therefore, additional studies are needed to confirm the diagnostic and predictive value of the pattern of immune response in TBI. While cytokine levels were found to be linked to the manifestation of neuropsychological symptoms, these associations were marginally significant and failed to pass the multiple testing correction. One reason for this is that multiple factors influence neuropsychological symptoms including environmental factors and psychological state (49, 50) as well as genetic factors and innate biological variability (51, 52). In addition, cytokines interact with other factors to activate complex downstream signaling networks (53). Future studies should focus on patterns of cytokine activity or expression in order to elucidate their network-level activity and function.

There were some limitations to this study. Firstly, we observed marked changes in serum cytokine levels during acute phase; a time course of 1 week may not have been sufficient to observe the actual dynamics of cytokines profiles. Secondly, serum cytokine levels do not directly reflect the CNS environment. Cerebrospinal fluid (CSF) is considered to provide data that are more reliable in this regard, but the collection of CSF samples involves lumbar puncture, which is poorly tolerated by patients. Finally, a longer follow-up time is needed to observe the full range of neuropsychological outcomes caused by inflammation-related cytokines in mTBI, especially given that macrophages and microglia remain active in the CNS for months or years post-injury and can adopt aberrant functions.

## Conclusions

The results of our study indicate that persistent, low-grade systemic inflammation exists in mTBI patients. Higher cytokines levels were associated with a greater severity of PCS and worse cognitive function in acute phase. In addition, elevated levels of specific cytokines in acute phase were positively associated with cognitive outcome in the chronic phase. Our findings demonstrate that serum cytokine measurements provide important information on post-mTBI outcome, additional studies are needed to clarify the pathological basis of the relationship between inflammation and neuropsychological symptoms of mTBI through combining serum cytokine measurements and neuroimaging approaches.

## Data Availability Statement

The datasets generated for this study are available on request to the corresponding author.

## Ethics Statement

The research procedures were approved by the Ethics Committee of the Second Affiliated Hospital of Wenzhou Medical University. All subjects provided written, informed consent prior to their participation in the study in accordance with the Declaration of Helsinki.

## Author Contributions

YS contributed to the design of the Luminex experiment, analyzed data, drafted, and revised the manuscript. XN, ZW, SW, SG, and CS prepared the experimental materials and analyzed data. BY, GB, and DZ recruited the subjects and collected serum samples. FZ, LB, and MZ contributed to study conception and design and revised the article. All authors read and approved the final manuscript.

### Conflict of Interest

The authors declare that the research was conducted in the absence of any commercial or financial relationships that could be construed as a potential conflict of interest.
