# Supplementary material for: Elevated Serum Levels of Inflammation-Related Cytokines in Mild Traumatic Brain Injury Are Associated With Cognitive Performance
Source: Front Neurol. 2019 Oct 23;10:1120. doi: 10.3389/fneur.2019.01120 (PMC6819507; doi:10.3389/fneur.2019.01120)
Supplement: Supplementary file 4 [file Table_1.DOCX]

**Supplementary Figure S1 |** Longitudinal changes in post-concussion symptoms and cognitive test performance in mTBI patients. Box graphs show PCS was significantly improved at T2 and T3, still severer than controls at T3. There is significantly recovery in the Trail Making A test and Digit Symbol Coding test at T2 and T3, as well as reach control levels at T2. Boxplots represent medians and interquartile (n = 52 for all three time points). Statistically significant differences between patients and controls at each time point and between different time points within patients are indicated by gray and black asterisks, respectively. * P < 0.016, ** P < 0.001.

**Supplementary Figure S2 |** Percentage changes from acute phase to follow-ups in post-concussion symptoms and cognitive test performance in mTBI patients. Boxplots represent medians and interquartile (n = 52 for both time points). TMA = Trail Making A test. DCS = Digital Symbol Coding test.

**Supplementary Figure S3 |** ROC curves of cytokines for distinguishing acute mTBI patients (n = 95) from controls.
