# Supplementary material for: Elevated Serum Levels of Inflammation-Related Cytokines in Mild Traumatic Brain Injury Are Associated With Cognitive Performance
Source: Front Neurol. 2019 Oct 23;10:1120. doi: 10.3389/fneur.2019.01120 (PMC6819507; doi:10.3389/fneur.2019.01120)
Supplement: Supplementary file 5 [file Table_2.DOCX]

**Supplementary Tables**

**Supplementary Table S1 |** Summary of demographic and clinical information for Cohort 1 and Cohort 2 mTBI patients, respectively.

|  | **Cohort 1** | **Cohort 2** |  |  |  |  |
| --- | --- | --- | --- | --- | --- | --- |
| **Demographic^a^** | **mTBI(n=52)** | **mTBI(n=43)** | **Controls(n=54)** | ***p_a_* value^b^** | ***p_b_* value^b^** | ***P_c_*value^b^** |
| Age in years | 35.42 ± 13.09 | 36.53 ± 14.51 | 35.74 ± 11.51 | 0.678 | 0.815 | 0.810 |
|  | (31.78-39.07) | (32.07-41.00) | (32.60-38.88) |  |  |  |
| Gender | 29M:23F | 26M:17F | 29M:25F | 0.644 | 0.848 | 0.542 |
| Education in years | 8.88 ± 3.85 | 8.05 ± 3.62 | 9.43 ± 4.14 | 0.332 | 0.334 | 0.059 |
|  | (7.81-9.96) | (6.93-9.16) | (8.30-10.55) |  |  |  |
| **Neuropsychological** **tests^a^** |  |  |  |  |  |  |
| Trail Making A | 59.42 ± 45.12 | 65.81 ± 47.31 | 41.70 ± 23.55 | 0.957 | **0.006** | **0.007** |
|  | (46.89-71.96) | (51.25-80.38) | (35.27-48.13) |  |  |  |
| Digit Symbol Coding | 35.52 ± 16.10 | 34.17 ± 15.70 | 46.93 ± 16.88 | 0.832 | **<0.001** | **<0.001** |
|  | (31.04-40.00) | (29.33-39.00) | (42.32-51.53) |  |  |  |
| Digit Span-forward | 7.88 ± 1.55 | 7.79 ± 1.52 | 8.33 ± 1.67 | 0.900 | 0.790 | 0.756 |
|  | (7.45-8.32) | (7.32-8.26) | (7.87-8.79) |  |  |  |
| Digit Span-backward | 3.73 ± 1.39 | 3.86 ± 1.30 | 4.51 ± 1.90 | 0.286 | 0.195 | 0.761 |
|  | (3.34-4.12) | (3.46-4.27) | (4.00-5.04) |  |  |  |
| Language Fluency | 17.19 ± 5.22 | 14.86 ± 4.85 | 18.93 ± 6.51 | 0.068 | 0.689 | **0.018** |
|  | (15.74-18.64) | (13.37-16.35) | (17.15-20.70) |  |  |  |
| **Symptoms severity^a^** |  |  |  |  |  |  |
| PCS | 10.38 ± 6.72 | 10.14 ± 7.95 | 2.33 ± 2.83 | 0.595 | <0.001 | <0.001 |
|  | (8.51-12.26) | (7.69-12.59) | (1.56-3.11) |  |  |  |
| **mTBI severity n (%)** |  |  |  |  |  |  |
| Loss of conscious | 47(90.4%) | 39(90.7%) | NA | 1.00 |  |  |
| Post traumatic amnesia | 6(11.5%) | 3(7.0%) | NA | 0.69 |  |  |
| GCS=15 | 52(100%) | 43(100%) | NA | - |  |  |
| GCS=13,14 | 0(0%) | 0(0%) | NA | - |  |  |
| **Causes for mTBI n (%)** |  |  |  |  |  |  |
| Acceleration/deceleration | 34(65.4%) | 26(60.5%) | NA | 0.62 |  |  |
| Ground level fall | 2(3.8%) | 5(11.6%) | NA | 0.29 |  |  |
| Fall from height | 4(7.7%) | 2(4.7%) | NA | 0.86 |  |  |
| Assaults | 11(21.2%) | 10(23.3%) | NA | 0.81 |  |  |
| Direct impact blow to head | 0(0.0%) | 1(2.3%) | NA | 0.92 |  |  |

^a^ Continuous variable are expressed as mean ± SD (90% CI) and categorical variables are expressed as frequency and percentage. Neuropsychological tests were presented with raw scores.

^b^ *p_a_* value for comparison between Cohort 1 and Cohort 2. *p_b_* and *p_c_* for Cohort 1 and Cohort 2 compared with controls respectively. Statistically significant P values are shown in bold.

Abbreviations: GCS, Glasgow Coma Scale; mTBI, mild traumatic brain injury; NA, non-available; PCS, Post Concussive Symptoms Scale.

**Supplementary Table S2 |** Serum cytokine levels of mTBI patients in Cohort 1 and Cohort 2 and controls.

| Cytokine(pg/ml) | Original | Replication | Total |  | Pooled |
| --- | --- | --- | --- | --- | --- |
| median (IQR) | mTBI (n = 52) | mTBI (n = 43) | mTBI (n = 95) | Control (n = 54) | mTBI vs. control  P value |
| CCL2 | **249.47(117.87) *** | **279.14(205.89) **** | 250.63(139.73) | 211.40(69.14) | **<0.001** |
| IL-1β | **2.85(0.68) *** | **3.22(1.68) **** | 2.98(1.01) | 2.58(1.19) | **<0.001** |
| IL-4 | 6.38(17.47) | 6.38(13.89) | 6.38(13.89) | 9.45(12.45) | 0.133 |
| IL-6 | **1.23(1.48) **** | **1.33(2.83) **** | 1.33(1.81) | 0.84(0.53) | **<0.001** |
| IL-8 | 8.67(6.17) | 9.95(7.66) | 8.97(6.73) | 7.56(3.55) | 0.061 |
| IL-10 | 0.56(0.70) | 0.38(1.03) | 0.44(0.84) | 0.45(0.80) | 0.588 |
| IL-12 | 20.81(14.66) | 24.74(21.31) | 22.25(15.77) | 20.81(12.23) | 0.087 |
| IFN-γ | 9.05(12.75) | 10.73(11.79) | 10.12(13.00) | 10.77(9.58) | 0.783 |
| TNF-α | 2.77(1.50) | 3.20(2.41) | 2.68(1.78) | 2.58(1.56) | 0.197 |

*P < 0.005, ** P < 0.001 for Cohort 1 and Cohort 2 comparison with controls separately. The P values with statistical significance are bold.
